# Artificial Ivory

**Published:** 1883-01

**Authors:** 


### Artificial Ivory.

It is said that artificial ivory of a pure white color and very durable has been manufactured by dissolving shellac in ammonia, mixing the solution with oxide of zinc, driving off ammonia by heating, powdering, and strongly compressing in moulds.

